# Supplementary material for: Conformational Rearrangements Regulating the DNA Repair Protein APE1
Source: Int J Mol Sci. 2022 Jul 20;23(14):8015. doi: 10.3390/ijms23148015 (PMC9324194; doi:10.3390/ijms23148015)
Supplement: Supplementary file 1 [file ijms-23-08015-s001.zip › ijms-1787753-supplementary.pdf]

Conformational rearrangements regulating the DNA repair protein APE1

Nina Komaniecka[1], Marta Porras[1,2], Louis Cairn[1], J. Ander Santas[1,2], Nerea Ferreiro[1,2], J. Carlos Penedo[3,4] and Sonia Bañuelos[1,2]*

[1] Biofisika Institute (UPV/EHU, CSIC), University of the Basque Country (UPV/EHU), Leioa, Spain
[2] Department of Biochemistry and Molecular Biology, University of the Basque Country (UPV/EHU), Leioa, Spain
[3] Centre of Biophotonics, Laboratory for Biophysics and Biomolecular Dynamics, Scottish Universities Physics Alliance (SUPA) School of Physics and Astronomy, University of St. Andrews, St. Andrews KY16 9SS, United Kingdom
[4] Centre of Biophotonics, Laboratory for Biophysics and Biomolecular Dynamics, Biomedical Sciences Research Complex, School of Biology, University of St. Andrews, St. Andrews KY16 9ST, United Kingdom
* Correspondence: sonia.banuelos@ehu.es; Tel.: +34 94 601 3347

Supplementary Material

Fig. S1

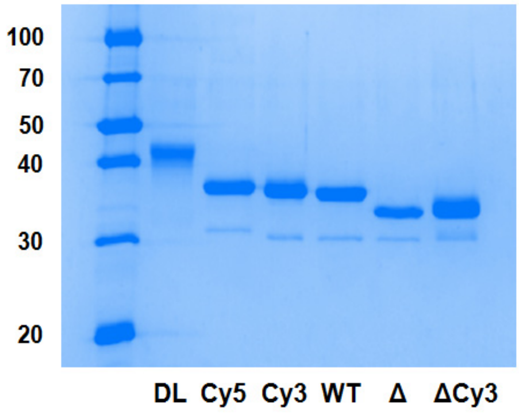

Figure S1: SDS-PAGE of the various APE1 variants. Full-length forms: Doubly labelled with Cy3 and Cy5 (DL), with Cy5, with Cy3 and unlabelled protein (WT). ΔN33-APE1 truncated forms: Unlabelled (Δ) and labelled with Cy3 (ΔCy3). MW markers sizes are indicated in KDa.

Fig. S2

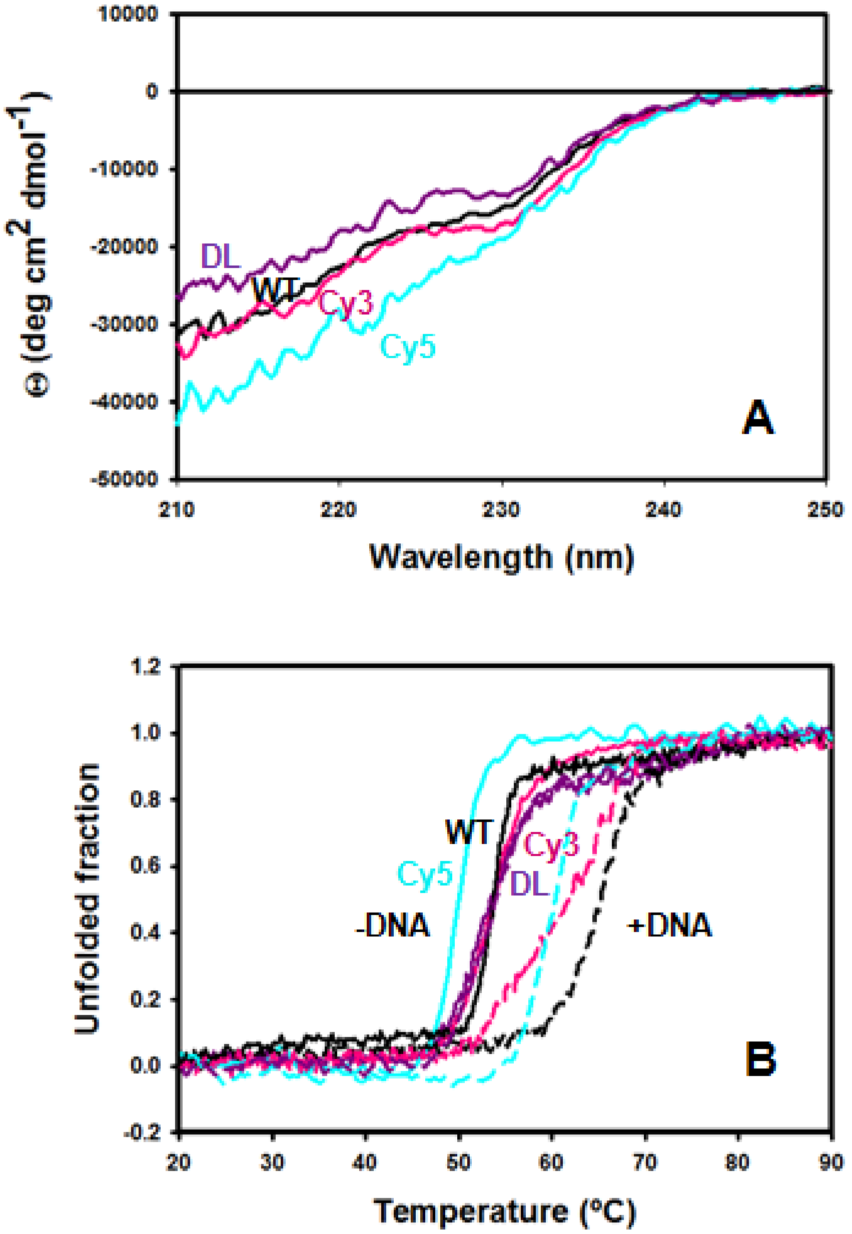

Figure S2: (A) Far-UV spectra of the different APE1 variants: unlabelled (black), Cy3-APE1 (magenta), Cy5-APE1 (cyan) and doubly labelled (purple). The protein concentration was 4 μM in buffer 20 mM potassium phosphate pH 7.0, 50 mM NaCl, 5 mM $MgCl_2$. **(B)** Thermal denaturation profiles as based on the change in ellipticity at 222 nm in the absence (solid line) and presence (broken line) of an equimolar amount of product DNA. The scan rate was 1ºC / min.

Fig. S3

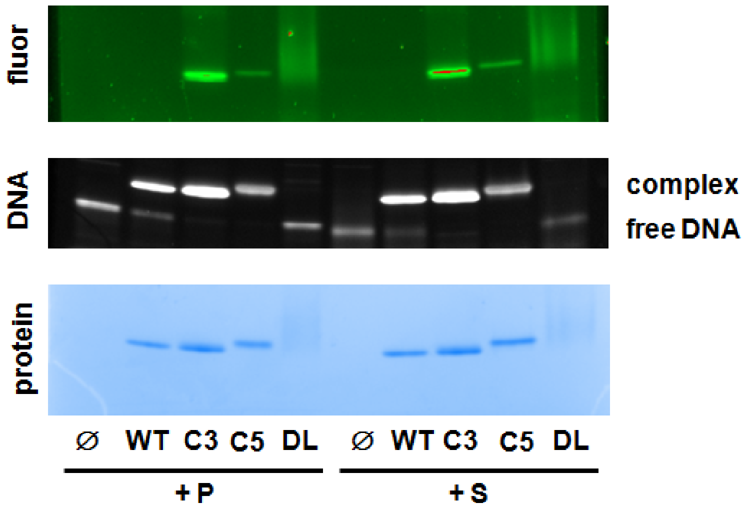

Figure S3: Binding of labelled APE1 to the oligonucleotides mimicking the abasic product (P) and substrate (S). Samples with 2 μM DNA alone (∅) or equimolar mixtures with unlabelled (WT), Cy3-labelled (C3), Cy5-labelled (C5) or doubly labelled (DL) APE1 were analyzed by native gel electrophoresis. Bands were visualized based on their intrinsic fluorescence ("fluor"), stained with Gel-Red ("DNA") and Coommassie ("protein").

Fig. S4

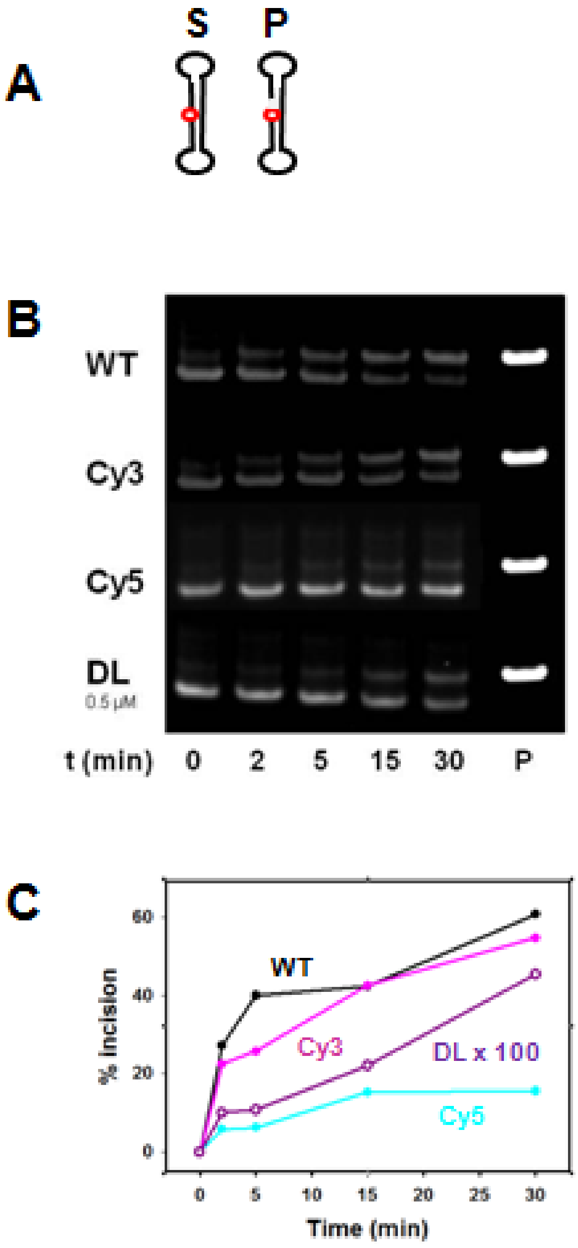

Figure S4: Incision kinetics of 2 μM substrate DNA by the various APE1 variants as followed by 18% polyacrylamide-urea gel electrophoresis. **(A)** Scheme of the substrate (S) and product (P) model oligonucleotides. **(B)** Gel of the samples at different reaction times, stained with GelRed. Unlabelled protein (WT), labelled with Cy3, Cy5, or doubly labelled with Cy3, Cy5 (DL). The concentration of the enzyme was 5 nM except for DL (0.5 μM). Migration of product DNA (P) is also shown. **(C)** Plot of the quantified band intensities as a function of time.

Fig. S5

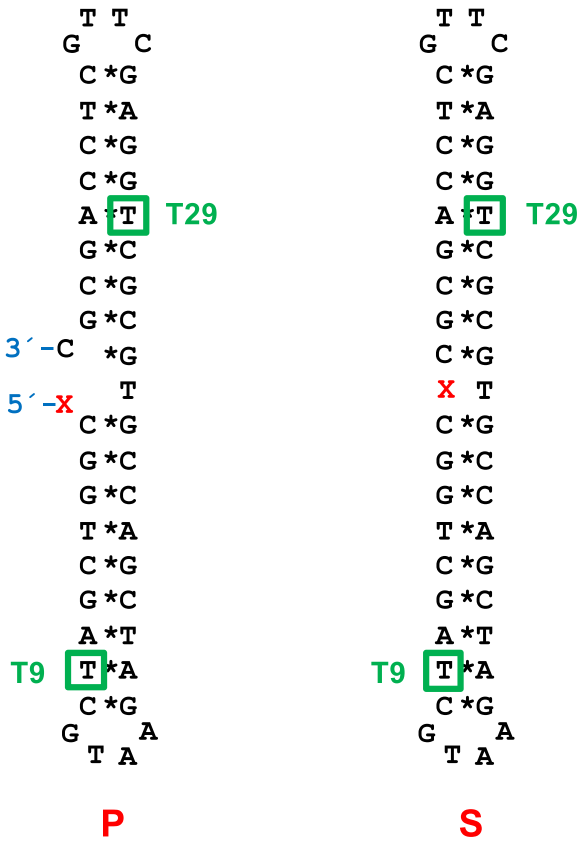

Figure S5: Sequence of the oligonucleotides used as model of the abasic APE1 product (left) and substrate (right), highlighting the two labelled thymines. X stands for tetrahydrofurane.

Fig. S6

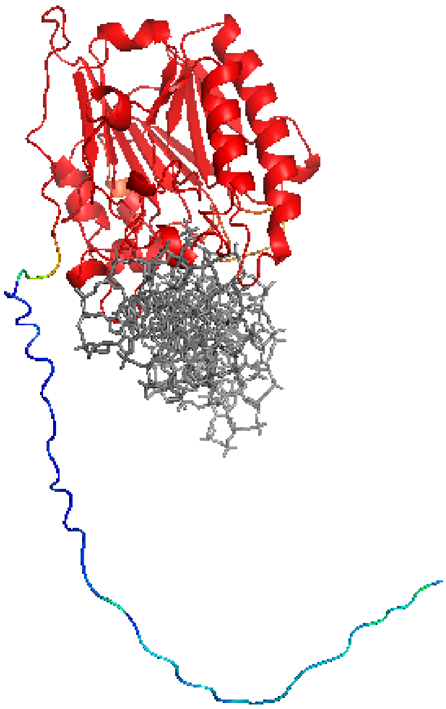

Figure S6: Original prediction as obtained with AlphaFold of human APE1 (UniProt entry P27695), represented as a cartoon, and aligned to the DNA part (grey sticks) of crystal structure 5DFF [17]. The colour code of the protein indicates the prediction reliability, from very high (red) to very low (blue) per-residue confidence score (pLDDT).

Fig. S7

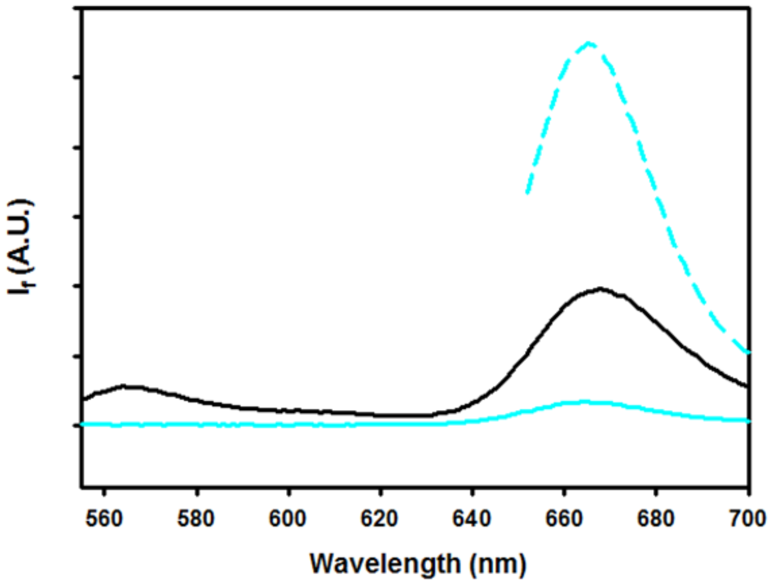

Figure S7: Emission spectra of doubly labelled APE1 (black line) and Cy5-APE1 (cyan solid line) upon excitation at 547 nm. Spectrum of Cy5-APE1 with λ_{exc} 647 nm (cyan broken line).

Fig. S8

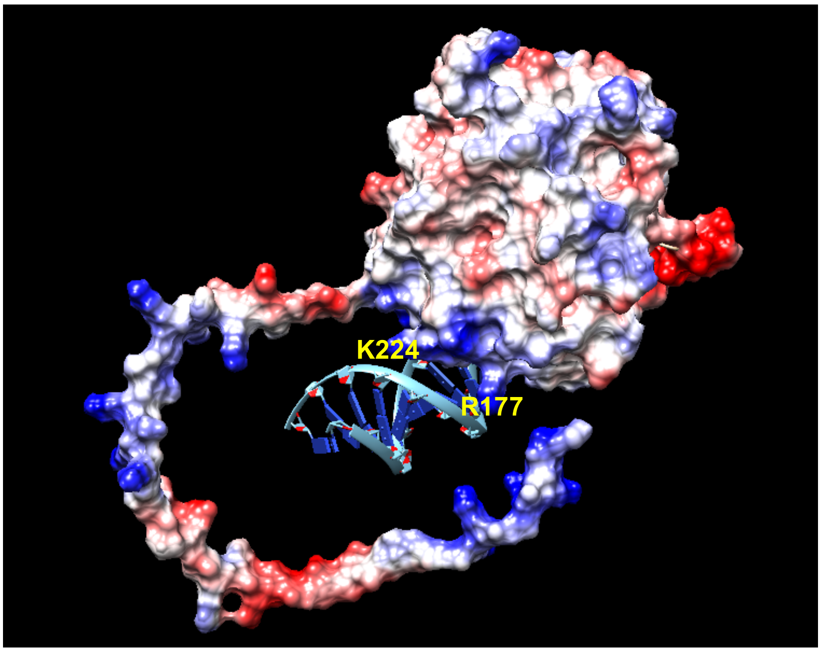

Figure S8: Charge distribution in full-length APE1 bound to DNA. The N-terminal 42 aa fragment was modelled on the structure of the APE1 / DNA product complex (5DFF [16]) based on FRET data. The protein is shown as a surface with "Coulombic colouring". Two positively charged residues are marked. The figure was prepared with Chimera.
